# BPTF promotes the progression of distinct subtypes of breast cancer and is a therapeutic target

**DOI:** 10.3389/fonc.2022.1011173

**Published:** 2022-11-30

**Authors:** Vladimir Bezrookove, Imran A. Khan, Mehdi Nosrati, James R. Miller, Sean McAllister, Altaf A. Dar, Mohammed Kashani-Sabet

**Affiliations:** California Pacific Medical Center Research Institute, San Francisco, CA, United States

**Keywords:** BPTF, triple-negative, ER-positive, breast cancer, oncogene

## Abstract

**Purpose:**

To assess the biomarker and functional role of the chromatin remodeling factor, bromodomain PHD finger transcription factor (BPTF), in breast cancer progression.

**Methods:**

*BPTF* copy number was assessed using fluorescence *in situ* hybridization. BPTF expression was regulated in breast cancer cells by shRNA/siRNA-mediated gene silencing and *BPTF* cDNA overexpression. The effects of regulating BPTF expression were examined on key oncogenic signaling pathways and on breast cancer cell proliferation, apoptosis, and cell cycle progression, as well as in xenograft models. The consequences of pharmacological bromodomain inhibition, alone or in combination with other targeted agents, on breast cancer progression were assessed in culture and in xenograft models.

**Results:**

*BPTF* copy number was gained in 34.1% and separately amplified in 8.2% of a breast cancer tissue cohort. Elevated *BPTF* copy number was significantly associated with increasing patient age and tumor grade and observed in both ER-positive and triple-negative breast cancer (TNBC) subtypes. *BPTF* copy number gain and amplification were also observed in The Cancer Genome Atlas (TCGA) breast cancer cohort. Stable shRNA-mediated silencing of *BPTF* significantly inhibited cell proliferation and induced apoptosis in TNBC and ER-positive human breast cancer cell lines. BPTF knockdown suppressed signaling through the phosphoinositide 3 kinase (PI3K) pathway, including reduced expression of phosphorylated AKT (Ser473), phosphorylated GSK-β (Ser9), and CCND1. These findings were confirmed following transient BPTF knockdown by a distinct siRNA in TNBC and ER-positive breast cancer cells. Stable suppression of BPTF expression significantly inhibited the in vivo growth of TNBC cells. Conversely, *BPTF* cDNA overexpression in TNBC and ER-positive breast cancer cells enhanced breast cancer cell proliferation and reduced apoptosis. *BPTF* targeting with the bromodomain inhibitor bromosporine, alone or in combination with the PI3K pathway inhibitor gedatolisib, produced significant anti-tumor effects against TNBC cells *in vitro* and *in vivo*.

**Conclusion:**

These studies demonstrate BPTF activation in distinct breast cancer subtypes, identify pathways by which BPTF promotes breast cancer progression, and suggest BPTF as a rational target for breast cancer therapy.

## Introduction

Breast cancer is the leading cause of cancer death in women, with an estimated 290,180 new cases and 43,250 deaths in 2022 (1). Breast cancer is a heterogeneous disease with multiple subtypes that vary with respect to marker expression and therapeutic response profiles. The expression of estrogen receptor (ER), progesterone receptor (PR) and human epidermal growth factor receptor 2 (HER2) are the main subtyping parameters in clinical use. Among the different subtypes, triple-negative breast cancer (TNBC) is highly aggressive, associated with a poor prognosis, and lacks effective targeted therapeutic options. Thus, there is an unmet need to identify new biomarkers as well as therapeutic targets for this breast cancer subtype.

Chromatin-remodeling factors are critical components of the machinery that controls gene expression. ATP-dependent chromatin-remodeling factors are classified into four major subfamilies (ISWI, SWI/SNF, CHD and INO80) based upon sequence homology of the associated ATPase (2). Nucleosome remodeling factor (NURF), a key ISWI family member (3), exists across all eukaryotic species and mediates some of its cellular functions through interaction with sequence-specific transcription factors (4, 5). BPTF (bromodomain PHD finger transcription factor), the largest subunit of the NURF chromatin-remodeling complex (4), plays an essential role in embryonic development (5) and in ATP-dependent chromatin remodeling (6). The human *BPTF* gene is located on chromosome 17q24, which is presumed to contain oncogenic elements given the demonstration of chromosomal gains in this locus in breast and other tumors (7–10). Comparative genomic hybridization analysis has shown frequent gains in the 17q22-q24 region in breast tumors (11–13). However, to date, the precise role played by BPTF in breast cancer progression is incompletely understood. In this study, we assess the biological role of BPTF in human breast cancer. We report substantial copy number gain of *BPTF*, assess the functional consequences of *BPTF* gene silencing in distinct subtypes of breast cancer, and explore the therapeutic consequences of bromodomain inhibition *in vivo*.

## Material and methods

### Cell culture

MDA-MB-231, MDA-MB-436 and MCF-7 human breast cancer cell lines were purchased from ATCC and authenticated by them (Manassas, VA). All cells were grown at 37°C in an atmosphere containing 5% CO_2._ MDA-MB-231 and MDA-MB-436 were grown in RPMI 1640 containing 5% FBS and 1X pen/strep, whereas MCF-7 cells were grown in DMEM containing 10% FBS and 1X pen/strep. Transient transfections were carried out by Lipofectamine-2000 (Thermo Fisher Scientific, South San Francisco, CA) according to the manufacturer’s protocol. All cell lines were confirmed as mycoplasma negative using the MycoFluor Mycoplasma Detection Kit (Thermo Fisher Scientific).

### Transfections and generation of stable transformants

Plasmids pCMV6-BPTF, pCMV6-Entry, control siRNA and BPTF-specific siRNAs were purchased from Origene (Origene Technologies, Rockville, MD) and used for transient transfection studies (performed as previously described (14) with effects on gene expression, cell cycle, and apoptosis assessed at various time points (24-72 hr) following transfection. The Lentiviral pLKO1-based shRNA vector targeting human *BPTF* was purchased from GE Dharmacon (Lafayette, CO), and used for transfection into various breast cancer cell lines along with its control (luciferase shRNA) as previously described (14). Stable transformants were generated by cloning selected shRNAs into the pLKO1-vector and co-transfection into 293T cells along with expression vectors containing the GAG/POL, REV and VSVG genes. Lentiviruses were harvested 48 hr after transfection. Sub-confluent human breast cancer cells were infected with harvested lentiviruses in the presence of 8 µg/ml of polybrene and were selected in 1µg/ml of puromycin at 48 hr post-infection in their respective culture medium.

### Quantitative real-time polymerase chain reaction (qPCR) analysis

Gene expression was assessed as previously reported (15, 16). mRNAs were assayed using the TaqMan Gene Expression Assays according to the manufacturer’s instructions (Thermo Fisher Scientific, South San Francisco, CA) and as described (15, 16). TaqMan probes for *BPTF* and *HPRT1* were purchased from Thermo Fisher Scientific.

### Colony formation assay

For the colony formation assay, 500-1000 cells were plated in a 6-well plate and allowed to grow until visible colonies appeared. Then, they were stained with crystal violet (Sigma, St Louis, MO) and counted. Cell cycle analysis and apoptosis were performed as described (17). Muse Annexin V and Dead Cell Assay Kit and Muse Cell Cycle Assay Kit (EMD Millipore, Billerica MA) were used per the manufacturer’s instructions.

### Western analysis

Western analysis was performed as described previously (15, 16). Target proteins were detected by using specific antibodies against BPTF (A300-973A at 1:1000 dilution) (Bethyl Laboratories, Montgomery, TX), pAKT (Ser473, #9271 at 1:500 dilution), total AKT (#4685, at 1:500 dilution), pGSK-3β (Ser9, #9323 at 1:500 dilution), CCND1 (#2978 at 1:500), BCL2 (#15071 at 1:500) (Cell Signaling Technology, Danvers, MA) and GAPDH (AB2302 at 1:10000 dilution, EMD Millipore, Billerica, MA).

### Tissue arrays

The tissue microarray for breast cancer samples was purchased from US Biomax Inc. (Rockville, MD). The TCGA breast cancer copy number dataset consisted of 960 samples, with available information regarding *BPTF* copy number for 114 triple negative samples and 512 ER-positive samples. Copy number data was obtained from cBioportal and the analyses are based on GISTIC or RAE algorithms (18).

### Fluorescent *in situ* hybridization

Fluorescent *in situ* hybridization was performed using BAC clones RP11-1134M2, RP11-29C18 and CTD-2314M10 to detect the *BPTF* locus on 17q24.3, as well as RP11-18L18 mapping to 17p11.1 as the centromeric probe for chromosome 17 (February 2009 freeze of the UCSC Genome Browser, http://genome.ucsc.edu). All clones were obtained from the Children’s Hospital of Oakland Research Institute (CHORI). BAC DNA was prepared with the Large-Construct kit (Qiagen, Valencia, CA) and labeled by nick translation with Alexa Fluor 488 and 594 dUTP’s (Thermo Fisher Scientific) as described (19). The quality and mapping of all probes was verified by hybridization to normal metaphase spreads in combination with a commercially available centromeric probe for chromosome 17 (Empire Genomics, Buffalo, NY) before tissue analysis. Hybridization on tissue sections was performed as described previously (19). Images were acquired using a Zeiss Axio Imager Z2 equipped with 63X objective and controlled by Axiovision software (Zeiss, Jena, Germany). The FISH signals were assessed and counted manually from images with collapsed Z stack layers. A minimum of 30 nuclei were evaluated from each case and the signals were interpreted according to guidelines described previously (20), and recorded as 1 to 10, with the number 20 assigned to those cases with signals that were too numerous to count. The individual assessing *BPTF* copy number was blinded to the identity and prognostic features of the cases analyzed.

### Cell cycle analysis

At least 1X10^6^ cells were used to perform cell cycle analysis. Cells were fixed with 70% ice cold ethanol while slowly mixing the cells followed by incubation for 3 hr at -20°C. The fixed cells were centrifuged at 300X g for 5 min and stained using Muse Cell Cycle Assay Kit (EMD Millipore, Billerica, MA). After incubation for 30 min at RT in dark, cell cycle profile of at least 10,000 events was obtained using Muse Cell Analyzer (EMD Millipore, Billerica, MA) according to the manufacturer. The cell cycle profiles were analyzed using the onboard Muse software. After adjusting the thresholds for cell size index, to exclude the debris, DNA content profiles were gated for G0/G1, S and G2/M cells. The percentage of cells in each gated population was used to perform statistical analysis.

### Cell viability analysis

At least 1X10^6^ cells were used to perform cell cycle analysis. Cells were incubated with Muse Annexin V and Dead Cell Kit (EMD Millipore, Billerica, MA) for 20 min at RT. The samples were then analyzed using Muse Cell Analyzer (EMD Millipore, Billerica, MA) and based on the measured intensity from Annexin V and 7-amino-actinomycin (7-AAD), the percentage of live, early apoptotic, late apoptotic and dead cells was determined using the onboard muse software.

### Fluorescence microscopy

Quantification of protein expression using immunofluorescence was performed on cells cultured on coverslips as previously described (21, 22). Antibodies against BPTF, pAKT (Ser473), total AKT, CCND1, and BCL2 were used to detect expression of individual proteins. Images were taken at fixed exposures with a Zeiss Axio Imager Z2 microscope and the fluorescence intensities of individual cells were quantified using Zeiss AxioVision Software. The mean pixel intensities were used for statistical analysis using Microsoft Excel and GraphPad Prism software. The expression data were quantified as amount of fluorescence per single nucleus.

### Animal studies

For the *BPTF* shRNA *in vivo* study, 1x10^6^ MDA-MB-231 cells were injected into the mammary fat pad of nude mice (nu/nu, 44 days old, female) (n=8 per group) (Charles River, Wilmington, MA). Cells were mixed with Matrigel (1:1) and injected in a total volume of 25 µl.

### Pharmacological studies

All drugs (including bromosporine and gedatolisib) were purchased from Selleck Chemicals. For the drug studies (bromosporine and/or gedatolisib), 1x10^6^ MDA-MB-231 cells were injected into the mammary fat pad of nude mice (*nu*/*nu*, 44 days old, female). Once tumors were palpable, mice were randomized and divided into the following treatment groups with average tumor volumes of 100-150 mm^3^: vehicle (n=6), bromosporine (n=6), gedatolisib (n=6), and bromosporine and gedatolisib combination (n=10). The animals were randomly assigned to the treatment groups, with the investigator performing tumor measurements blinded to the identity of the treatment groups. All drugs were administered intraperitoneally (i.p.) at the following doses: bromosporine (40 mg/kg) and gedatolisib (10 mg/kg). Bromosporine was administered five times a week, whereas gedatolisib was administered once weekly. Tumors were measured by caliper and volumes were calculated as a product of (length x width x width)/2.

### Statistical methods

Statistical analyses were applied as described (23). In the FISH analysis, the statistical significance of differences in *BPTF* copy number between distinct subgroups of breast cancer patients was assessed using Mann-Whitney, Fisher exact, and Chi-square tests. In the functional analysis of BPTF in breast cancer cell lines, statistical significance was determined by the Student’s t-test. To test for synergism, the combination index (CI) was calculated using Compusyn (Paramus, NJ) where CI <1, = 1 and > 1 indicates synergism, additive effect and antagonism, respectively, as previously described (24, 25). All quantified data represent an average of at least triplicate samples or as indicated. Error bars represent standard error of the mean. Two-tailed p values <0.05 were considered significant. The difference in expression of target proteins in different cells, as assessed by quantitative immunofluorescence, was tested for significance using the Kolmogorov–Smirnov test.

## Results

**Figure 1:**
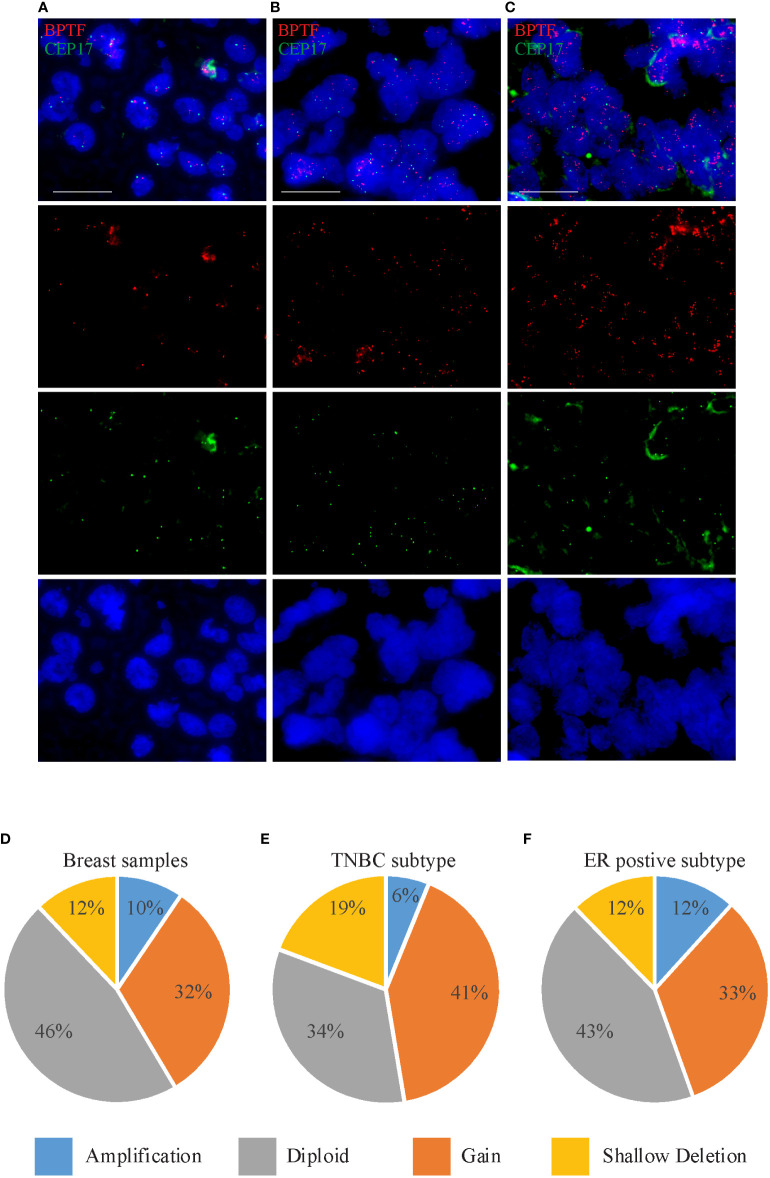
*BPTF* copy number gain in breast cancer samples. **(A-C)** Representative images of FISH analysis of breast tissue samples showing euploidy **(A)**, gain **(B)** and amplification **(C)** in *BPTF* copy number. **(D-F)** *BPTF* copy number in all breast cancer samples **(D)**, as well as TNBC **(E)** and ER-positive subtypes **(F)** from TCGA database. Scale bar 20μm.

Given the presence of copy number gains of 17q in human breast cancer (7), we assessed *BPTF* copy number using a previously developed assay (14) in a tissue microarray cohort (N=85) of primary breast cancer specimens (**Figures 1A-C** and **Table S1**). We observed a wide range of mean copy number for *BPTF*, from 1.78 to 7.82. In addition, there were five cases with obvious amplified signals (**Figure 1C**), where signal counting was impossible. We also recorded the percentage of cells harboring 3 or more copies of *BPTF*. Elevated *BPTF* copy number (defined as mean copy number ≥3) was present in 34.1% of the cases of this cohort. In addition to copy number gains, amplifications of the *BPTF* gene, defined as the ratio of *BPTF* to chromosome 17 centromeric probe > 2, were detected in 8.2% of the cohort (7/85 cases). Initially, we assessed the correlation between *BPTF* copy number and certain clinical or histologic variables. There was a significant association between *BPTF* copy number and patient age. The mean *BPTF* copy number was significantly higher in patients greater than 49 years old versus those less than 49 years old (mean of 4.69 vs. 2.92; P<0.01, Mann-Whitney test). Correspondingly, the percentage of cells with 3 or greater copies of *BPTF* was significantly higher in older than younger patients (mean of 46.5% vs. 28.9%; P<0.01, Mann-Whitney test). In addition, there was a significant association between *BPTF* copy number and tumor grade, both as assessed by mean copy number (P<0.05, Fisher exact test) and by percentage of cells with at least 3 copies of *BPTF* (P<0.02, Chi-square test). Intriguingly, each of the five high-grade tumors harbored greater than 2.50 copies of the *BPTF* gene.

We then assessed the association between *BPTF* copy number and molecular marker expression (i.e., ER/PR/HER2 status). There was a significant association between *BPTF* copy number and ER status. The mean copy number was 5.28 in ER-positive cases *versus* 2.76 in ER-negative cases (P<0.01, Mann-Whitney test). In addition, the percentage of cells with 3 or more copies of *BPTF* was significantly higher in ER-positive *versus* ER-negative cases (mean of 45.5% *vs*. 31.7%; P<0.01, Mann-Whitney test). Using similar analyses, no significant associations were identified between *BPTF* copy number and either HER2 or TNBC status.

Next, we analyzed *BPTF* copy number in the publicly available TCGA dataset using cBioportal software (18). *BPTF* copy number was available for 960 samples, consisting of 114 TNBC and 512 ER-positive samples. The *BPTF* gene was amplified in 9.47% of the entire cohort, including 6.14% of TNBC and 11.7% of ER-positive cases (**Figures 1D-F**). Furthermore, *BPTF* copy number gain was observed in 31.9% of the entire cohort, including 41.2% of TNBC and 32.8% of ER-positive cases (**Figures 1D-F**). These data demonstrate evidence of *BPTF* copy number gain in different breast cancer subtypes, and specifically in TNBC and ER-positive subtypes. As a result of these observations, we focused our attention on the functional effects of *BPTF* on the progression of TNBC and ER-positive human breast cancer subtypes.

**Figure 2:**
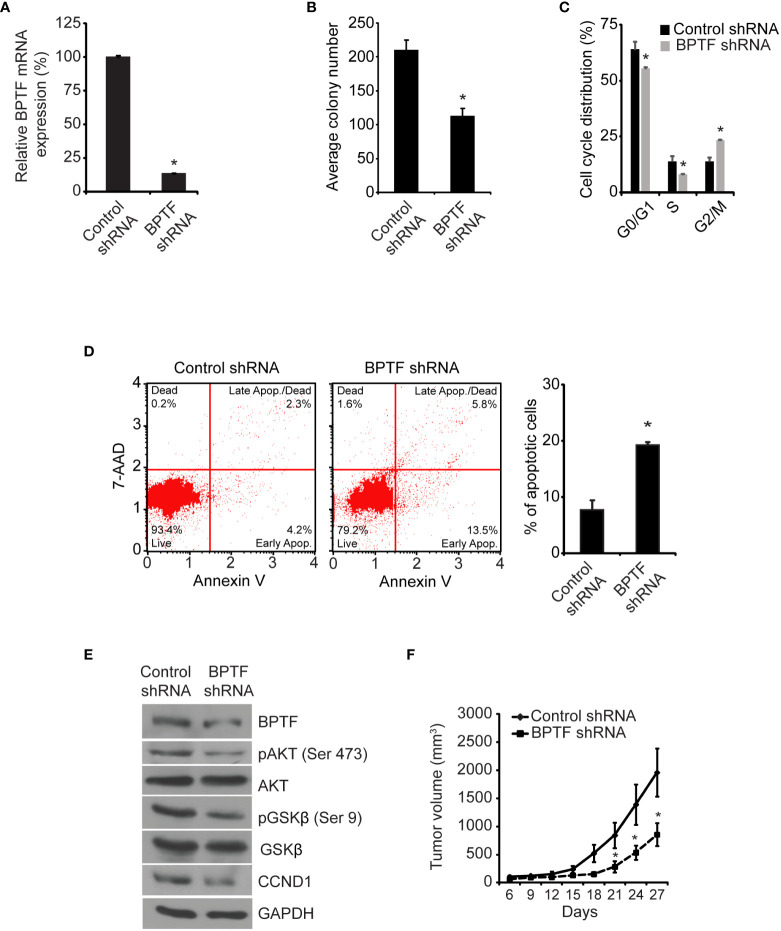
Effects of shRNA-mediated suppression of *BPTF* on MDA-MB-231 cells. **(A)** *BPTF* mRNA levels following shRNA-mediated suppression. **(B)** Mean colony number after BPTF suppression. **(C)** Cell cycle phases following BPTF knockdown. **(D)** Analysis of apoptotic rate after *BPTF* silencing based on detection of 7-AAD and Annexin V. **(E)** Western analysis of expression of various proteins following shRNA-mediated *BPTF* knockdown. **(F)** *In vivo* tumor cell growth following shRNA-mediated suppression of *BPTF*. *p < 0.05.

Initially, we assessed the consequences of regulation of BPTF expression using stable expression of a well-characterized shRNA targeting human *BPTF* (14). Stable shRNA-mediated *BPTF* knockdown (**Figure 2A**) significantly suppressed the colony formation ability of TNBC MDA-MB-231 cells (hereafter, referred to as 231) when compared to non-specific shRNA (control shRNA)-expressing cells (**Figure 2B**). BPTF suppression led to a significant decrease in the S-phase cell population when compared to control shRNA-expressing cells (**Figure 2C** and **Supplementary Figure 1A**). A significant induction in apoptosis was observed in BPTF knockdown cells *versus* control shRNA-expressing cells (**Figure 2D**). We then assessed the consequences of regulation of BPTF expression on the PI3K signaling pathway, which is of particular significance to breast cancer progression (26, 27). *BPTF* silencing suppressed expression of pAKT (Ser473), pGSK-β (Ser9) and CCND1 (**Figure 2E**) when compared to control shRNA-expressing cells. BPTF suppression significantly suppressed (by 57%) the *in vivo* tumor growth of 231 cells in the mammary fat pad (**Figure 2F**).

**Figure 3:**
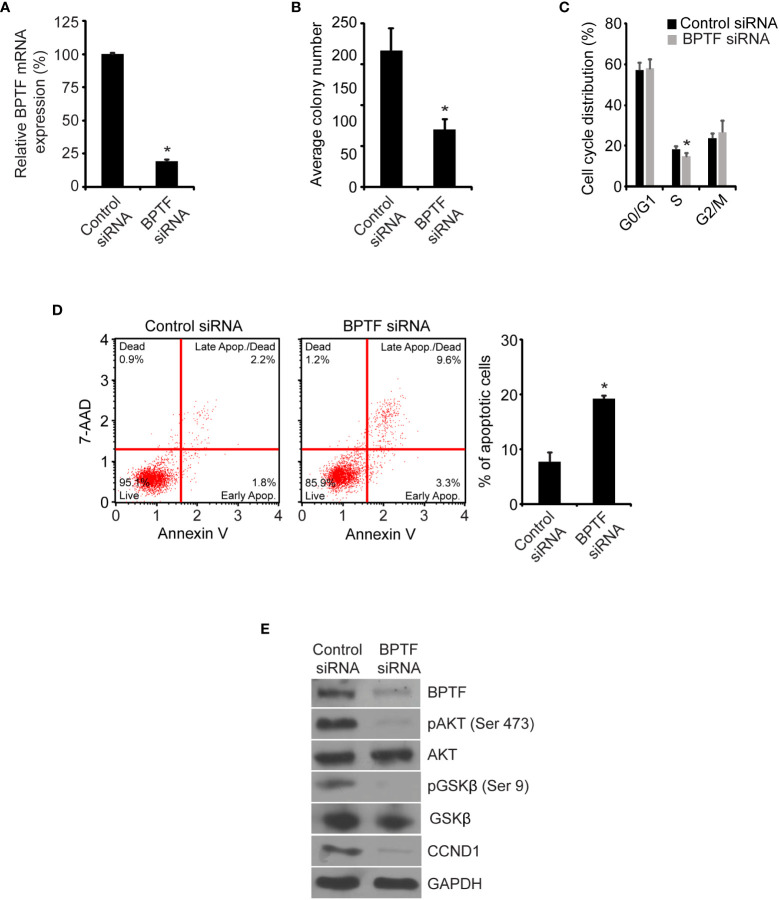
Effects of siRNA-mediated suppression of BPTF on MDA-MB-231 cells. **(A)** *BPTF* mRNA expression after siRNA knockdown. **(B)** Mean colony number after *BPTF* knockdown. **(C)** Cell cycle analysis following siRNA-mediated suppression of *BPTF*. **(D)** Analysis of apoptotic rate after *BPTF* silencing based on detection of 7-AAD and Annexin V. **(E)** Western analysis of expression of various proteins following siRNA-mediated *BPTF* knockdown. *p < 0.05.

To confirm these observations, we assessed the effects of treatment of 231 cells with a siRNA targeting a distinct sequence on *BPTF* mRNA. Transient transfection of the anti-*BPTF* siRNA suppressed its expression (**Figure 3A**) and significantly reduced 231 cell colony formation ability (**Figure 3B**) when compared to transfection of 231 cells with a control siRNA. This was accompanied by a significant decrease in the percentage of cells in S-phase (**Figure 3C** and **Supplementary Figure 1B**), as well as a three-fold increase in apoptosis, following siRNA-mediated *BPTF* knockdown in 231 cells (**Figure 3D**). In addition, suppression of pAKT (Ser473), pGSK-β (Ser9) and CCND1 expression at the protein level was observed following treatment with a distinct anti-*BPTF* siRNA (**Figure 3E**).

**Figure 4:**
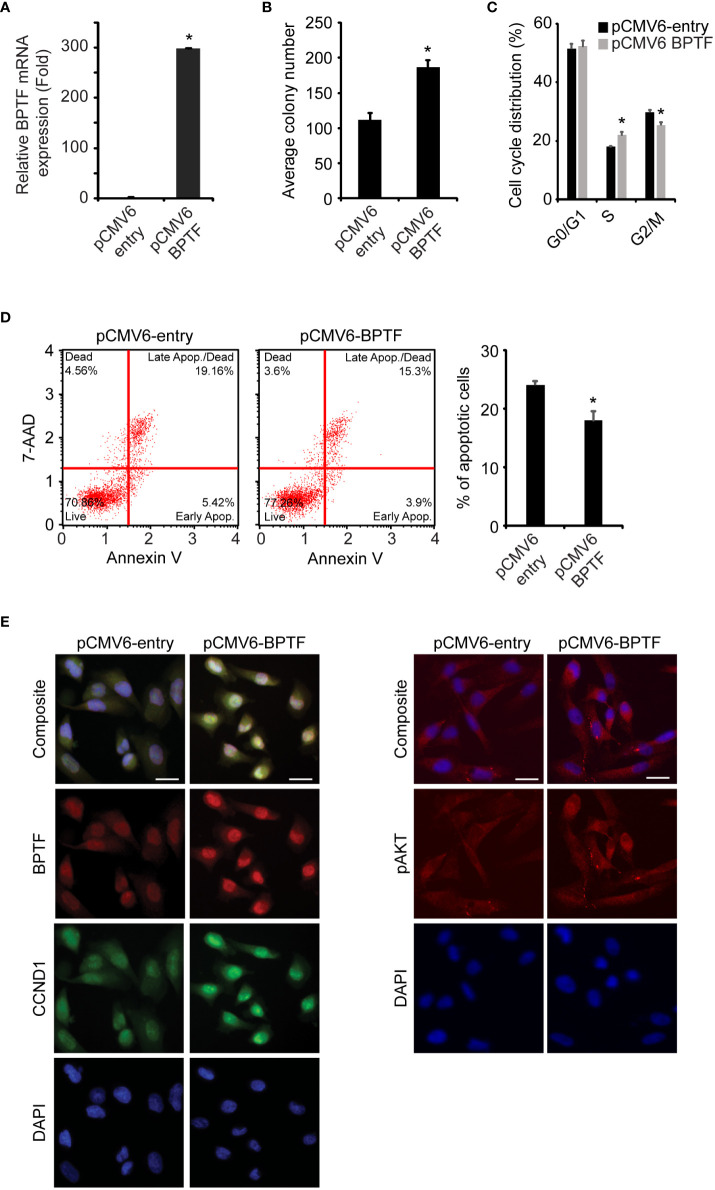
Effects of BPTF overexpression on MDA-MB-231 cells. **(A)** BPTF overexpression in MDA-MB-231 cells. **(B)** Colony formation assay following BPTF overexpression. **(C)** Cell cycle analysis following BPTF overexpression. **(D)** Analysis of apoptotic rate after BPTF overexpression based on detection of 7-AAD and Annexin V. **(E)** Quantitative immunofluorescence of various proteins following BPTF overexpression. Scale bar 20μm. *p < 0.05.

Next, we assessed the consequences of BPTF overexpression in 231 cells. Transient transfection of *BPTF* cDNA (**Figure 4A**) significantly increased 231 cell colony formation (**Figure 4B**), along with a significant increase in the S-phase population (**Figure 4C**, **S1C**) and significantly reduced apoptotic rate (**Figure 4D**). Immunofluorescence analysis showed significantly increased expression of pAKT (Ser473), CCND1, and BCL2 in 231 cells overexpressing BPTF compared to control vector-expressing cells (**Figure 4E** and **Supplementary Figures 1D-H**). These observations indicate that BPTF mediates important effects on breast cancer adhesion-independent cell proliferation, cell cycle progression, and apoptotic activity, in part through its activation of the PI3K pathway.

In addition, we assessed the consequences of regulation of BPTF expression in MDA-MB-436 (hereafter, 436), another TNB cell line, to further substantiate these findings. *BPTF* silencing, either by stable shRNA transduction (**Supplementary Figures 2A-E**), or by transient expression of a distinct siRNA (**Supplementary Figures 3A-E**), resulted in decreased colony formation potential, reduced population of cells in S-phase, and increased apoptotic rate. *BPTF* knockdown in 436 cells resulted in downregulation of PI3K pathway proteins. Overexpression of *BPTF* cDNA in 436 cells (**Supplementary Figures 4A-E**) led to enhanced colony formation, increased S-phase, and reduced apoptosis, along with increased expression of pAKT (Ser473), pGSK-β (Ser9), CCND1 and BCL2 (**Supplementary Figures 5A-E**). Taken together, these findings demonstrate the broad-based role of BPTF in promoting the progression of TNBC.

**Figure 5:**
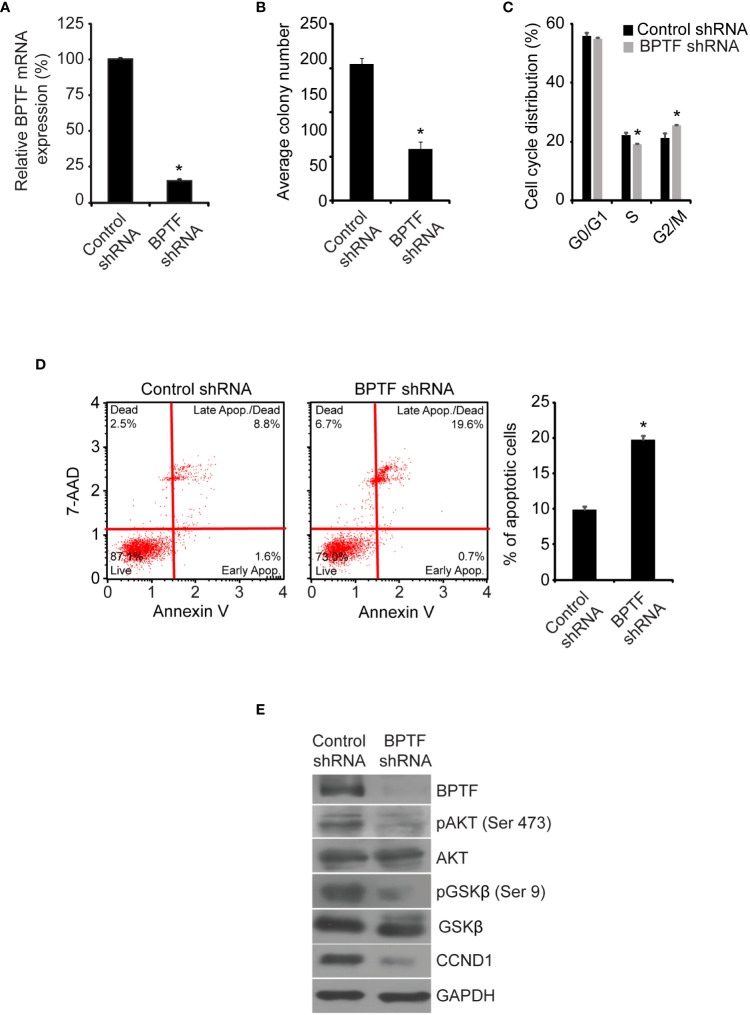
Effects of shRNA-mediated suppression of *BPTF* on MCF-7 cells. **(A)** *BPTF* mRNA expression following shRNA-mediated knockdown. **(B)** Colony formation ability of MCF-7 cells following *BPTF* silencing. **(C)** Cell cycle analysis following BPTF suppression. **(D)** Analysis of apoptotic rate after *BPTF* silencing based on detection of 7-AAD and Annexin V. **(E)** Western analysis of expression of different proteins following *BPTF* silencing in MCF-7 cells. *p < 0.05.

**Figure 6:**
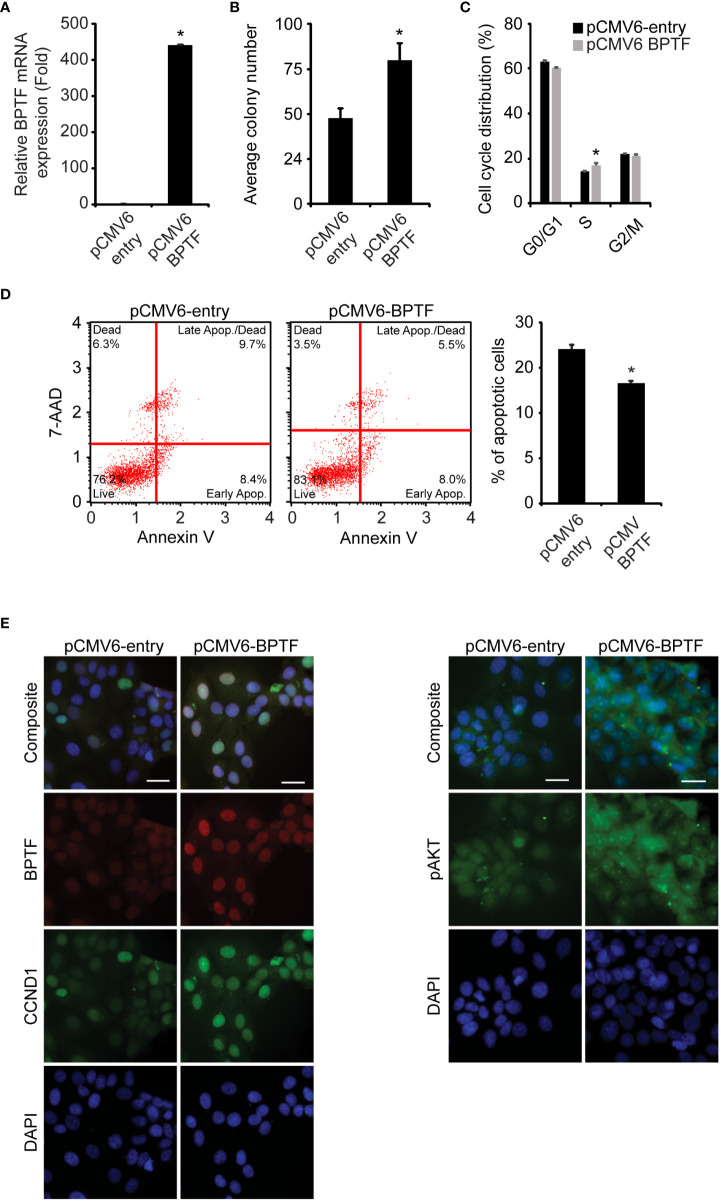
Effects of BPTF overexpression on MCF-7 cells. **(A)** BPTF overexpression in MCF-7 cells. **(B)** Colony forming capacity of MCF-7 cells after BPTF overexpression. **(C)** Cell cycle analysis following BPTF overexpression. **(D)** Analysis of apoptotic rate after BPTF overexpression based on detection of 7-AAD and Annexin V. **(E)** Quantitative immunofluorescence of various proteins following BPTF overexpression. Scale bar 20μm. *p < 0.05.

Tissue microarray and TCGA data also indicated a significant gain in *BPTF* copy number in ER-positive samples, prompting the investigation of its functional role in this subtype using the MCF-7 cell line. Stable shRNA-mediated silencing of *BPTF* (**Figure 5A**) suppressed MCF cell colony formation (**Figure 5B**), when compared to control shRNA-expressing cells. *BPTF* suppression in MCF-7 cells resulted in reduced proportion of cells in S-phase (**Figure 5C** and **Supplementary Figure 5F**), along with a significant increase in the apoptotic population (**Figure 5D**). We observed marked decreases in expression of pAKT (Ser473), pGSK-β (Ser9) and CCND1 (**Figure 5E**) upon BPTF knockdown, similar to that observed in TNBC cells. These results were confirmed following transient transfection of a distinct anti-*BPTF* siRNA in MCF-7 cells. Specifically, *BPTF* knockdown resulted in reduced colony formation, decreased S-phase, and induction of apoptosis, along with suppressing the expression of pAKT (Ser473), pGSK-β (Ser9) and CCND1 (**Supplementary Figures 6A-E**). In addition, overexpression of *BPTF* cDNA (**Figure 6A**) enhanced MCF-7 cell colony formation (**Figure 6B**). BPTF overexpression also increased the percentage of cells in S-phase (**Figure 6C** and **Supplementary Figure 5G**) and reduced the apoptotic index (**Figure 6D**). Immunofluorescence analysis showed increased expression of pAKT (Ser473) and CCND1 in BPTF-overexpressing cells (**Figure 6E** and **Supplementary Figures 7A-C**). Taken together, these results demonstrate a functional role for BPTF in promoting progression of both triple-negative and ER-positive breast cancer subtypes.

Finally, we aimed to develop a targeted therapeutic approach for breast cancer using the bromodomain inhibitor bromosporine, which has demonstrated affinity for BPTF (28). Bromosporine treatment produced cytotoxic effects against both 231 and MCF-7 cells in culture (**Supplementary Figure 8A**), with significant reduction in the S-phase population (**Supplementary Figures 8B, C**) and was accompanied by a significantly increased apoptotic rate (**Supplementary Figures 8D**, **E**). Bromosporine administration also resulted in significantly reduced expression of pAKT (Ser473) and pGSK-β (Ser9) in breast cancer cells (**Supplementary Figure 8F**). Thus, bromosporine treatment reproduced several key effects observed following *BPTF* gene silencing.

**Figure 7:**
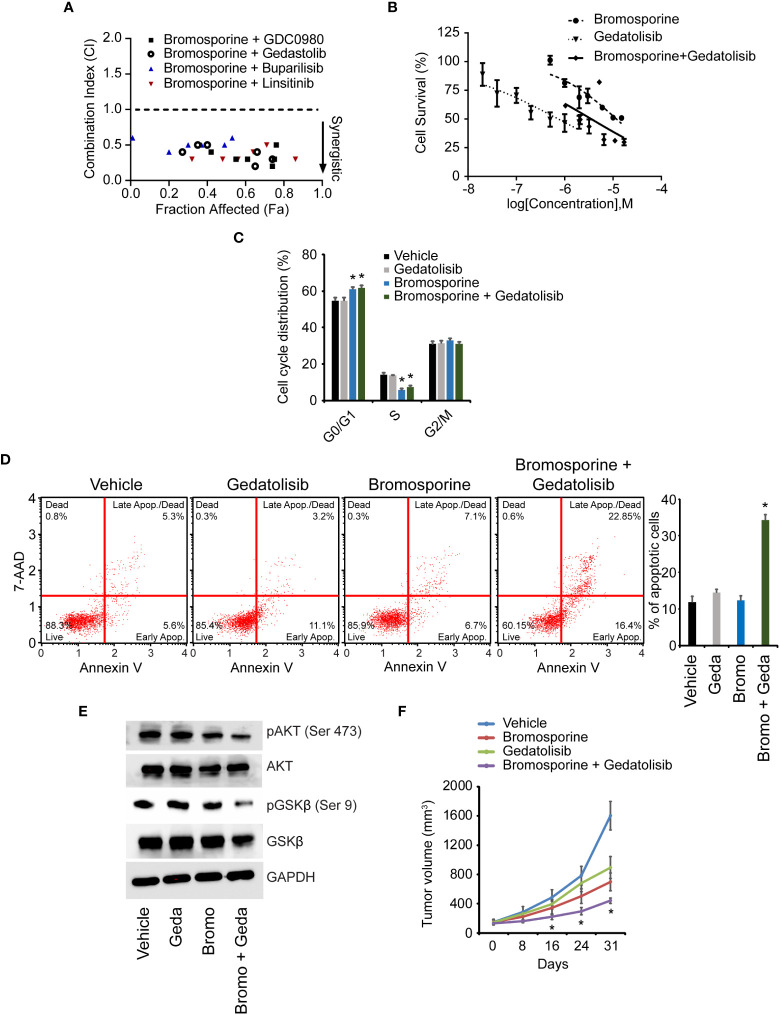
Effects of combinatorial therapy with bromosporine and PI3K pathway-targeting agents. **(A)** Combination index (CI) values for various bromosporine-containing combinations in 231 cells. **(B)** Effects of treatment with bromosporine and gedatolisib, alone or in combination, on viability of 231 cells. **(C)** Cell cycle analysis following treatment of 231 cells. **(D)** Analysis of apoptotic rate after drug treatment of 231 cells based on detection of 7-AAD and Annexin V. **(E)** Western analysis of expression of different proteins following drug treatment of 231 cells. **(F)** *In vivo* growth of 231 tumors following treatment with bromosporine and gedatolisib, alone or in combination. *p < 0.05.

Given the regulation of the PI3K pathway by BPTF, we aimed to determine the consequences of combinatorial therapy involving bromosporine and a panel of drugs that act on various targets within the PI3K pathway. We assessed synergy using the combination index (25), in which a score of <1 reveals synergy. Combinations of bromosporine and several PI3K-targeting agents showed synergistic activity in 231 cells (**Figure 7A**). We focused our attention on the combination of bromosporine and gedatolisib, an inhibitor of PI3K and mechanistic target of rapamycin kinase (MTOR). While both drugs were active against 231 cells alone, the combination improved efficacy (**Figure 7B**). In addition, treatment with the bromosporine/gedatolisib combination resulted in a significant decrease in the S-phase cell population (**Figure 7C** and **Supplementary Figure 8G**), accompanied by an increased apoptotic rate (**Figure 7D**), and downregulation of PI3K pathway signaling (**Figure 7E**). Finally, we assessed the *in vivo* efficacy of the bromosporine-gedatolisib combination in the 231 model. While treatment with either drug resulted in anti-tumor activity, combinatorial treatment was significantly more effective than either drug alone (**Figure 7F**).

## Discussion

BPTF is a chromatin remodeler and transcription factor, and plays important roles in histone acetylation, gene regulation and embryonic development (29, 30). BPTF-mediated ATP-dependent chromatin remodeling, directly coupled to H3K4 trimethylation, maintains *HOX* gene expression patterns during development (31). Previous studies showed that expression of BPTF played an important role in maintaining early mouse embryonic development and embryonic stem cell differentiation (5, 32), including the self-renewal capacity of mammary gland stem cells (33). The *BPTF* gene is localized to 17q24, a region reported to be amplified in breast cancer (7) and with copy number gains observed in other solid tumors (8, 9). However, to date, a pro-tumorigenic role for BPTF has not been described.

In this study, we report the functional and biological significance of BPTF in distinct breast cancer subtypes. We analyzed *BPTF* copy number in a tissue microarray cohort and identified copy number gains in 34.1% of cases, which significantly correlated with increasing patient age and tumor grade. Analysis of TCGA samples corroborated the *BPTF* copy number gain observed in breast cancer samples, including 41.2% of TNBC and 32.8% of ER-positive cases. These findings indicate that *BPTF* copy number is elevated in a substantial proportion of breast cancer specimens spanning different subtypes. Evidence of *BPTF* gene amplification in the TCGA cohort has been recently reported, along with an association between increased *BPTF* expression levels and reduced distant metastasis-free survival in ER-positive breast cancer (34). These results are consistent with analyses supporting activation of BPTF in other malignancies. We previously reported *BPTF* copy number gain in primary melanoma, along with a prognostic role (14). Higher *BPTF* expression levels have also been reported in hepatocellular (35) and colorectal cancers (36). Finally, BPTF overexpression was shown to predict poor prognosis in non-small cell lung cancer (37, 38).

Based on our analysis of the two tissue cohorts, we focused our functional studies on the triple-negative and ER-positive breast cancer subtypes. *BPTF* gene silencing, either using stable shRNA expression or transient siRNA transfection, significantly suppressed colony formation and induced apoptosis in both triple-negative and ER-positive breast cancer cell lines. Conversely, overexpression of *BPTF* cDNA resulted in increased colony formation and reduction in the apoptotic population in both TNBC and ER-positive cell lines. These observations suggest that BPTF regulates adhesion-independent proliferative capacity and apoptosis in distinct subtypes of breast cancer. The pro-proliferative role of BPTF in solid tumors has been reported by our group and others (14, 39). The current studies are consistent with these findings and emphasize BPTF’s important role in breast cancer progression. Orthotopic studies further validated the potent role played by BPTF in promoting tumor growth, as evidenced by decreased growth of 231 cells in the mammary fat pad following stable shRNA-mediated *BPTF* silencing.

To understand the mechanism by which BPTF promotes breast cancer progression, we investigated the effects of modulating BPTF expression on the PI3K pathway. We observed a significant role for BPTF in regulating the expression of pAKT (Ser473), pGSK-β (Ser9), CCND1, and BCL2, key members of this pathway. A high percentage of breast cancers are characterized by the constitutive activation of PI3Ks (40), linked to key hallmarks of tumorigenesis, including cell cycle progression, chemotherapeutic resistance, resistance to hypoxia and metastatic potential (26, 27). Due to the importance of PI3K signaling, pharmacological inhibitors of the pathway have been developed as anti-neoplastic agents (41), culminating in the FDA approval of alpelisib for ER-positive, HER2-negative, PIK3CA-mutant breast cancer (42). While our analysis focused on the activation of the PI3K pathway by BPTF, it is possible that BPTF exerts its pro-tumorigenic effects through additional signaling pathways beyond PI3K.

Our results are consistent with recent studies assigning a biological role to BPTF in various solid tumors, including a pro-invasive role in breast cancer (34). We previously reported a pro-oncogenic role for BPTF in melanoma by virtue of its modulation of the MAP kinase pathway as well as its promotion of resistance to targeted therapy (14). In addition, we showed that BPTF transduces certain key pro-proliferative effects mediated by the transcription factor MITF in melanoma (21). Separately, BPTF was shown to interact with c-MYC, thereby playing an important role in c-MYC-driven proliferation (39). And BPTF was reported to regulate the MAP kinase and PI3K pathways in lung adenocarcinoma (37) and epithelial-mesenchymal transition in colorectal cancer (35).

Chromatin remodeling factors represent a new class of therapeutic targets in cancer, given the development of bromodomain inhibitors to target the BET protein family (43). We observed that bromosporine was active in culture against both TNBC and ER-positive breast cancer lines. Given the regulation of the PI3K pathway by BPTF, we examined the combination of bromosporine with different PI3K pathway-targeting agents and identified synergistic activity for several such combinations. Specifically, the combination of bromosporine and gedatolisib showed significantly increased anti-tumor activity *in vivo* in the 231 model when compared to either agent alone. It is important to note that bromosporine targets different bromodomains and its anti-tumor activity may extend beyond BPTF targeting alone. Similarly, a recent study demonstrated that BPTF targeting can sensitize breast cancer cells to treatment with topoisomerase inhibitors (44).

## Data availability statement

The original contributions presented in the study are included in the article/**Supplementary Material**. Further inquiries can be directed to the corresponding authors.

## Ethics statement

The studies involving human participants were reviewed and approved by California Pacific Medical Center Institutional Review Board. The patients/participants provided their written informed consent to participate in this study. The animal study was reviewed and approved by California Pacific Medical Center Institutional Animal Care and Use Committe.

## Author contributions

Conceptualization, MK-S and AD. Methodology, VB, AD, SM. Software, JM. Formal analysis, VB, IK. AD, SM, JM. Investigation, VB, AD, SM. Resources, MN. Data curation, VB, IK, MN, AD, SM.Writing—original draft preparation, VB. AD, MS. Writing—review and editing, all authors. All authors contributed to the article and approved the submitted version.

## Funding

This work was supported by R01CA114337, R01CA215755 (MKS) and GRO Grant 280110008-0716 (AAD). We would like to acknowledge the California Pacific Medical Center Foundation for their support of the Cancer Avatar Program.

## Conflict of interest

The authors declare that the research was conducted in the absence of any commercial or financial relationships that could be construed as a potential conflict of interest.

## Publisher’s note

All claims expressed in this article are solely those of the authors and do not necessarily represent those of their affiliated organizations, or those of the publisher, the editors and the reviewers. Any product that may be evaluated in this article, or claim that may be made by its manufacturer, is not guaranteed or endorsed by the publisher.
