# Supplementary material for: BPTF promotes the progression of distinct subtypes of breast cancer and is a therapeutic target
Source: Front Oncol. 2022 Nov 30;12:1011173. doi: 10.3389/fonc.2022.1011173 (PMC9748419; doi:10.3389/fonc.2022.1011173)
Supplement: Supplementary file 1 [file DataSheet_1.pdf]

Supplementary Figure 1

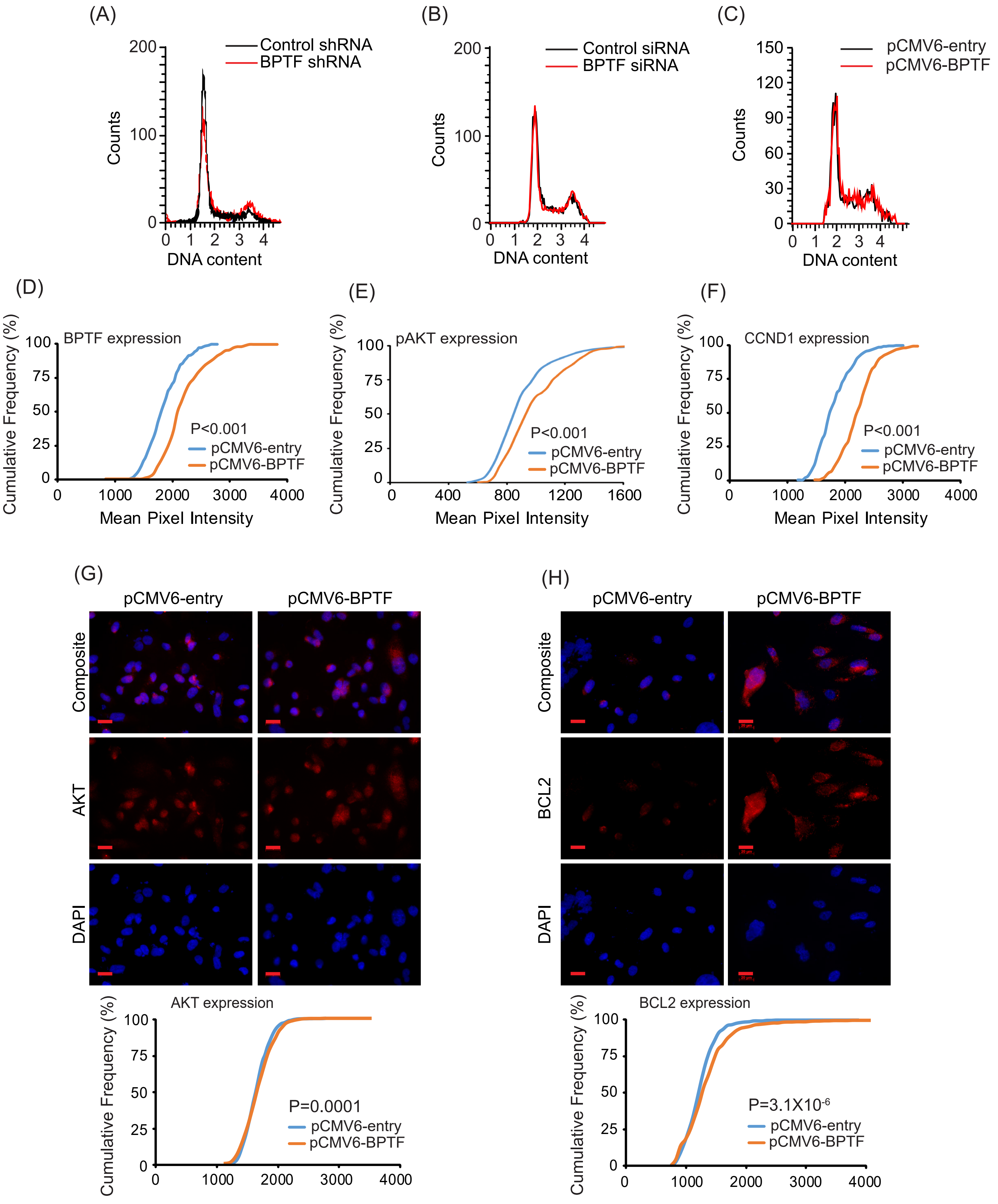

Supplementary Figure 1. **Effects of BPTF downregulation and overexpression on MDA-MB-231 cells**. (A-C) Cell cycle profiles following different methods employed to modulate expression of BPTF. (D-F) Analysis of BPTF, pAKT and CCND1 expression following the overexpression of BPTF. (G-H) Quantitative immunofluorescence analysis of total AKT and BCL2 following BPTF overexpression. Scale bar 20µm.

Supplementary Figure 2

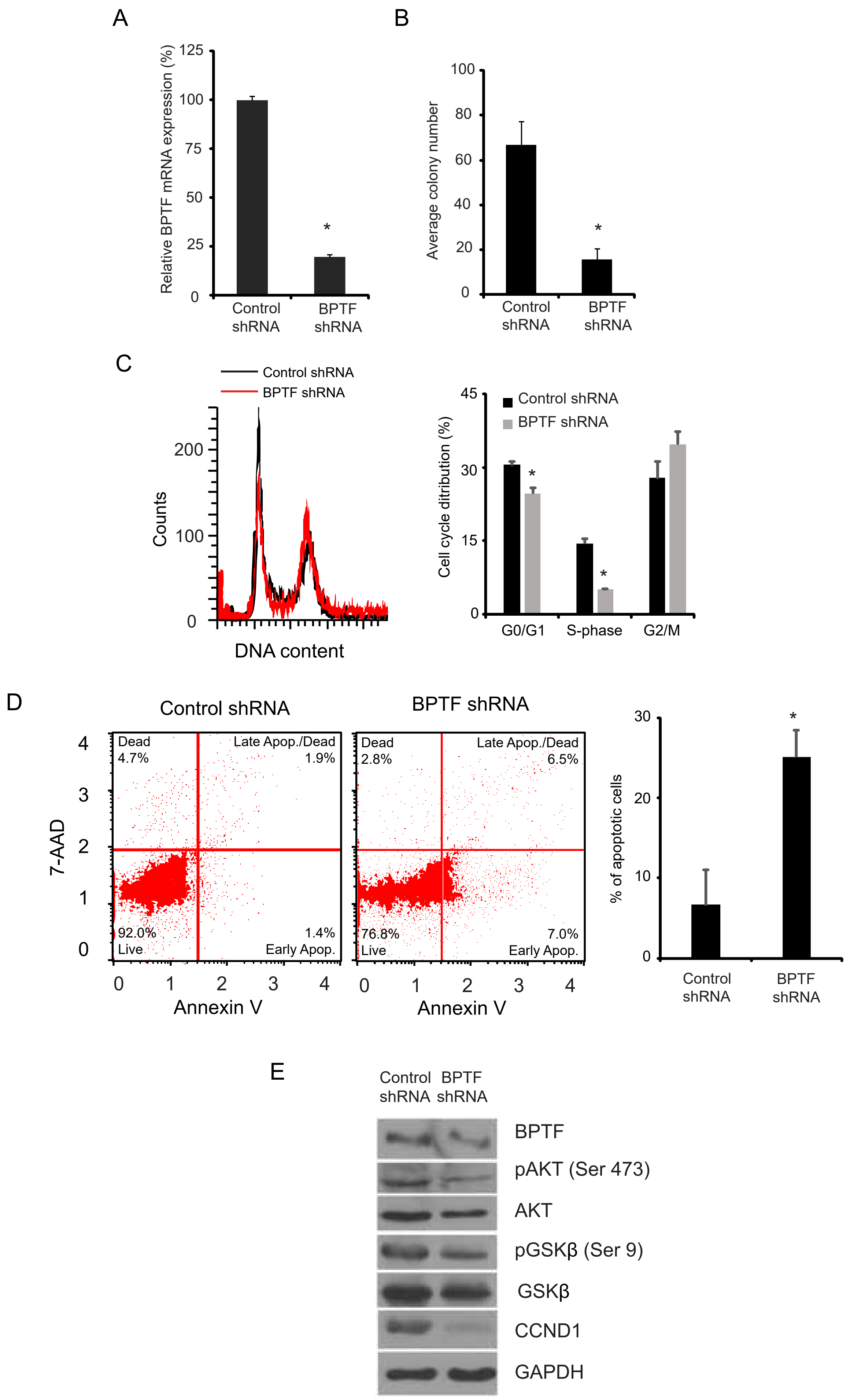

Supplementary Figure 2. Effects of shRNA-mediated suppression of *BPTF* on MDA-MB-436 cells. (A) BPTF mRNA expression after BPTF knockdown. (B) Colony formation analysis following anti-*BPTF* shRNA expression. (C) Cell cycle analysis following shRNA-mediated *BPTF* suppression. (D) Analysis of apoptotic rate after BPTF silencing based on detection of 7-AAD and Annexin V. (E) Western analysis of expression of various proteins following *BPTF* knockdown. * $p < 0.05$.

Supplementary Figure 3

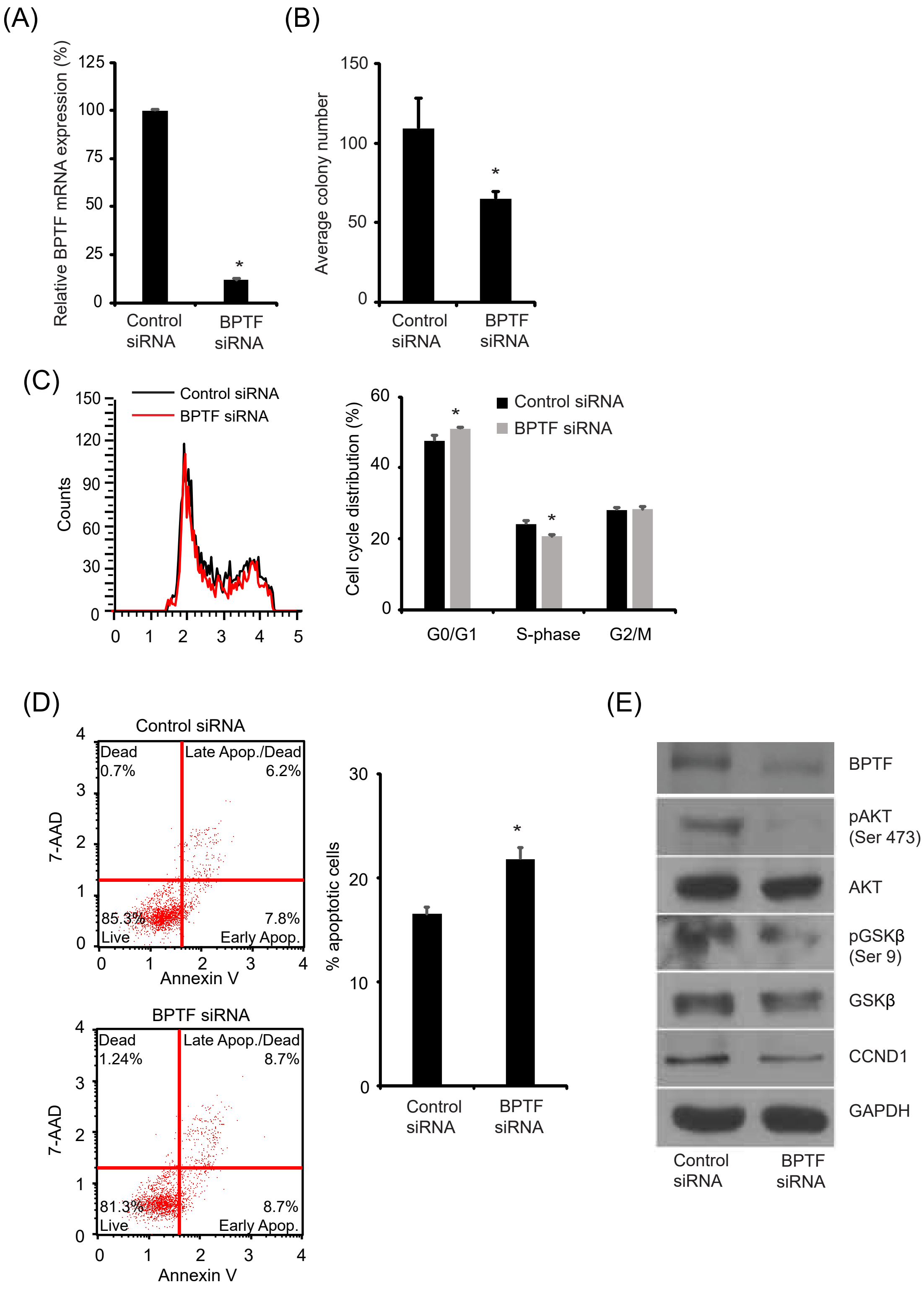

Supplementary Figure 3. Effects of siRNA-mediated suppression of *BPTF* on MDA-MB-436 cells. (A) *BPTF* mRNA expression following siRNA knockdown. (B) Colony formation ability following *BPTF* silencing. (C) Cell cycle analysis after BPTF knockdown. (D) Analysis of apoptotic rate after BPTF silencing based on detection of 7-AAD and Annexin V. (E) Western analysis of expression of various proteins following *BPTF* knockdown. * $p < 0.05$.

Supplementary Figure 4

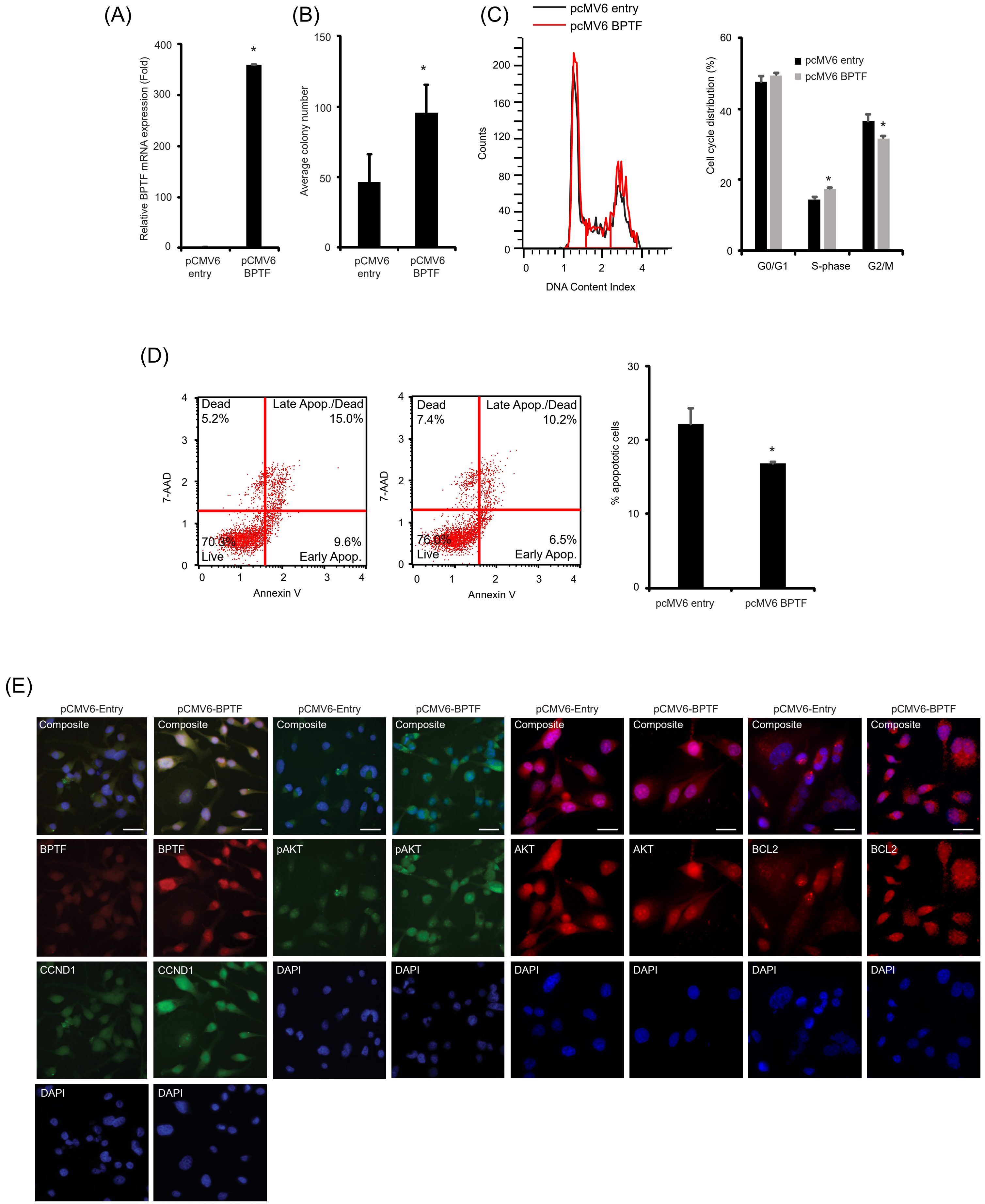

Supplementary Figure 4. Effects of BPTF overexpression on MDA-MB-436 cells. (A) *BPTF* mRNA analysis following BPTF overexpression. (B) Colony formation ability following BPTF overexpression. (C) Cell cycle analysis after BPTF overexpression. (D) Analysis of apoptotic rate after BPTF overexpression based on detection of 7-AAD and Annexin V. (E) Quantitative immunofluorescence of various proteins following BPTF overexpression. Scale bar 20µm. * $p < 0.05$.

Supplementary Figure 5

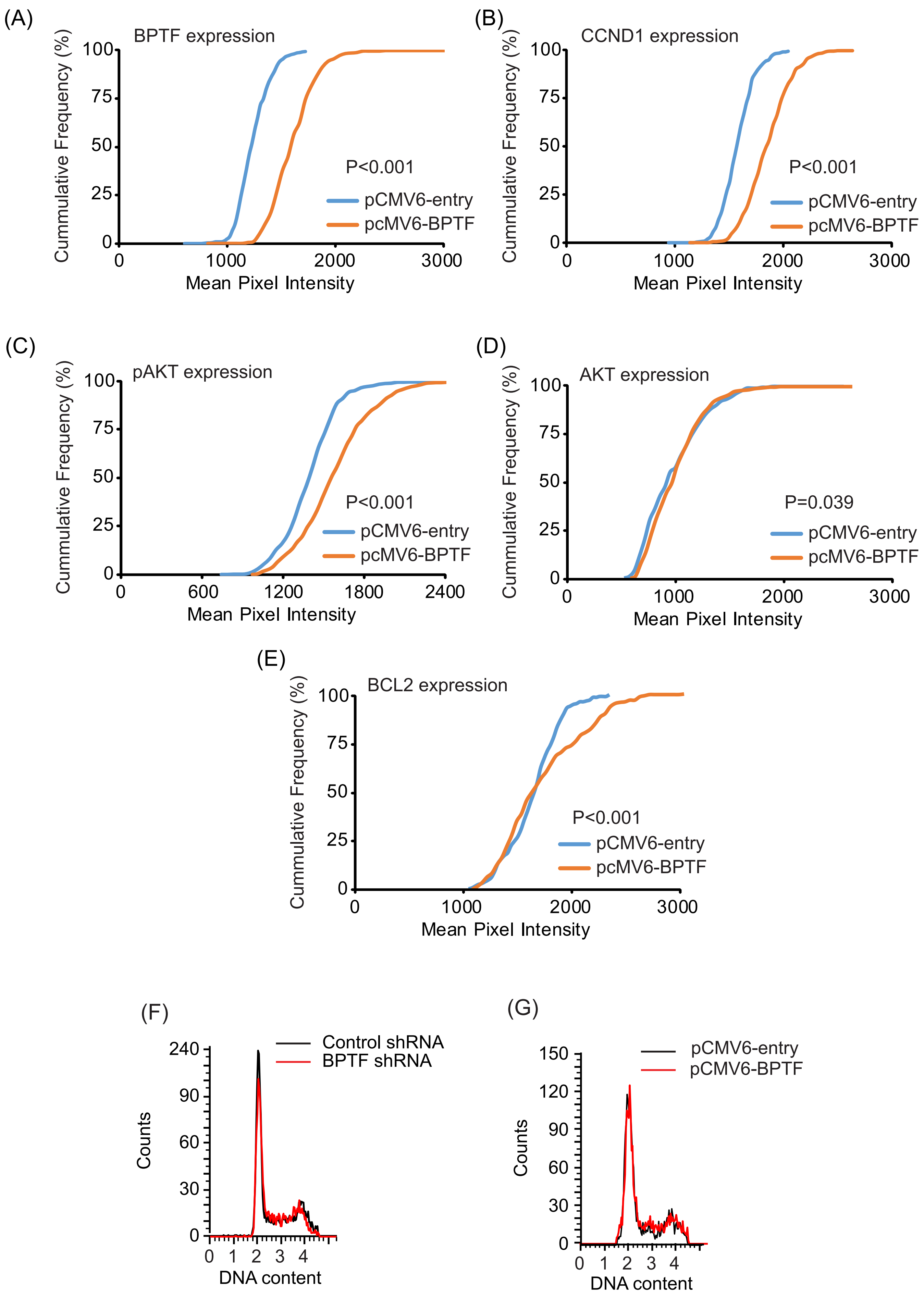

Supplementary Figure 5. Effects of BPTF overexpression on MDA-MB-436 cells. (A-E) Analysis of BPTF, CCND1, pAKT, AKT and BCL2 expression in MDA-MB-436 cells. (F) Cell cycle analysis of MDA-MB-436 after BPTF knockdown. (G) Cell cycle profile of MDA-MB-436 after BPTF overexpression.

Supplementary Figure 6

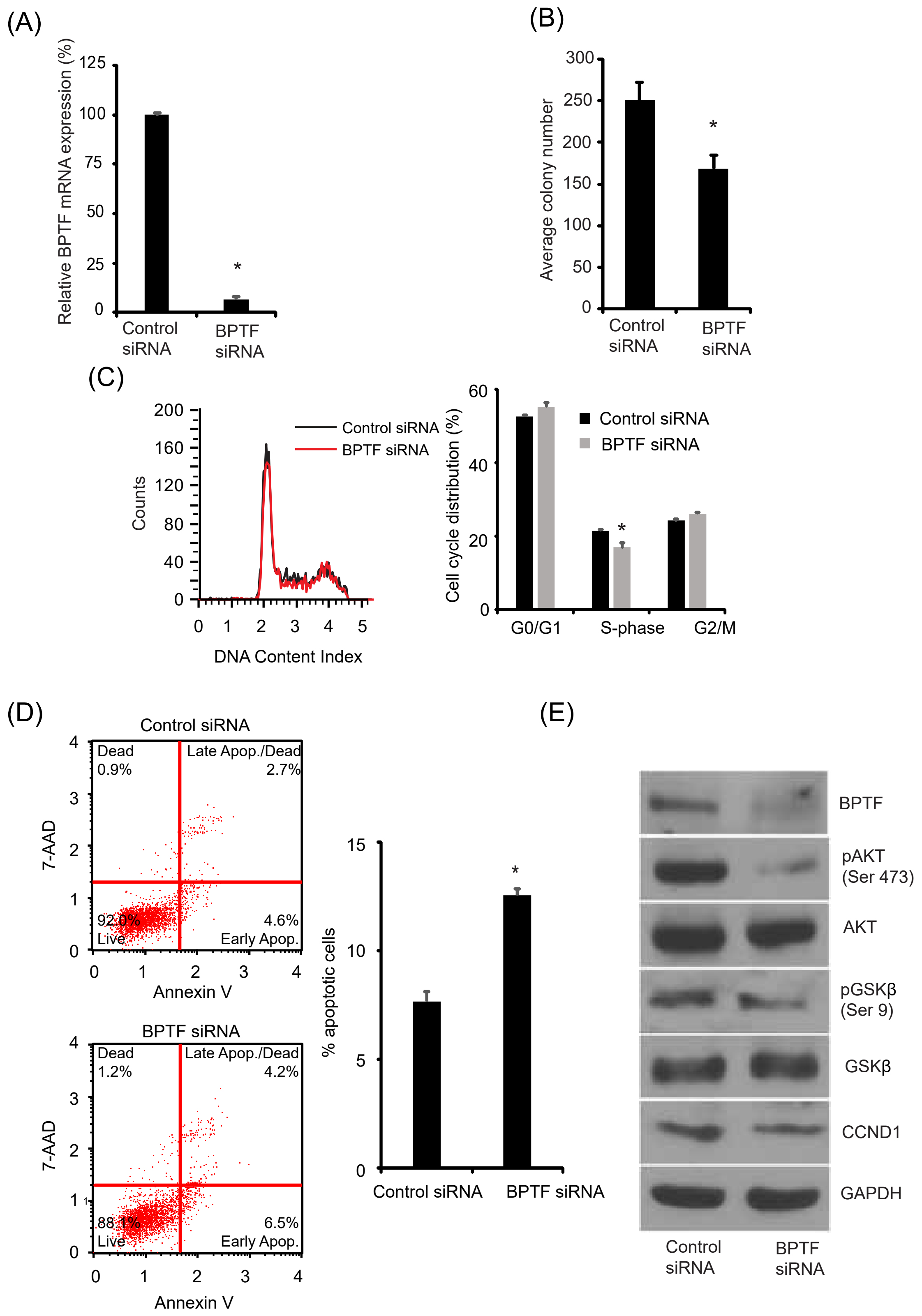

Supplementary Figure 6. Effects of siRNA-mediated suppression of *BPTF* on MCF-7 cells. (A) *BPTF* mRNA expression following siRNA knockdown. (B) Colony formation ability following BPTF suppression. (C) Cell cycle analysis after *BPTF* knockdown. (D) Analysis of apoptotic rate after BPTF silencing based on detection of 7-AAD and Annexin V. (E) Western blot analysis of expression of various proteins following *BPTF* knockdown. * $p < 0.05$.

Supplementary Figure 7

(A)

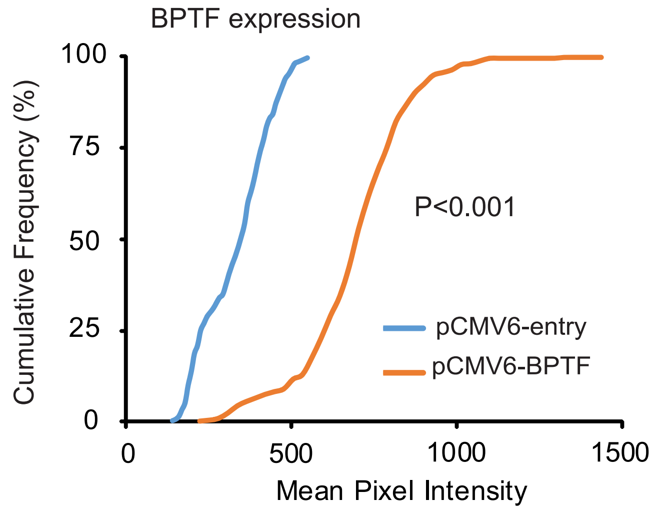

(B)

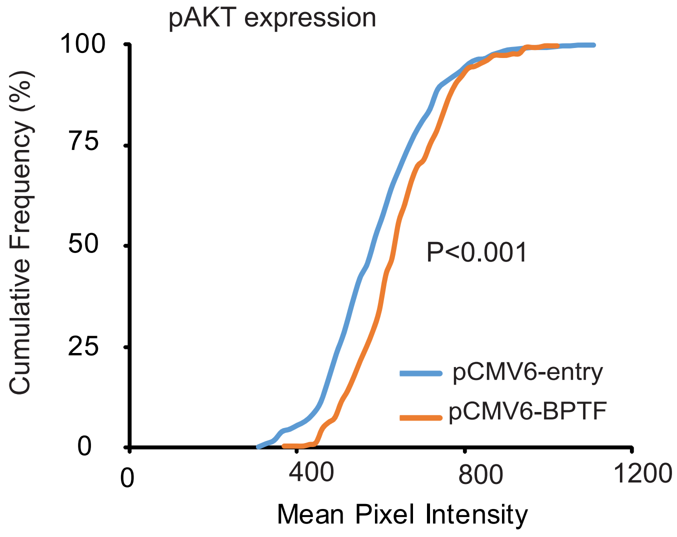

(C)

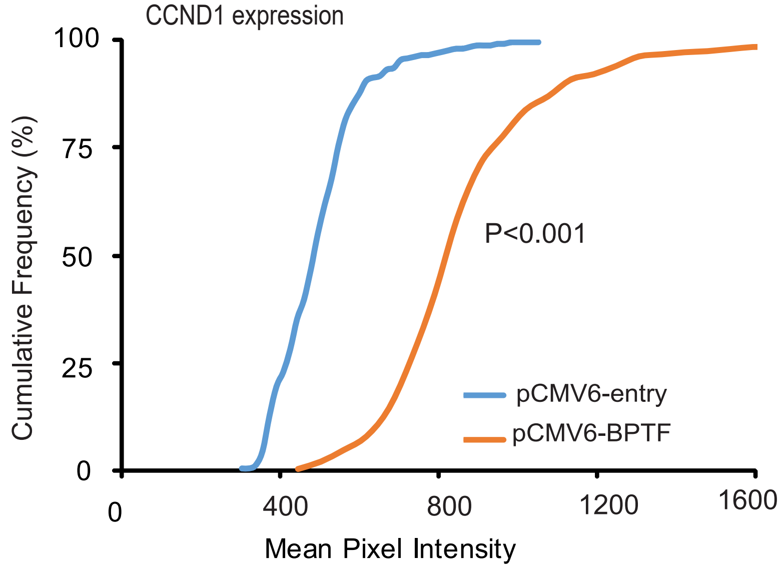

Supplementary Figure 7. Quantitative immunofluorescence analysis. (A-C) Analysis of BPTF, pAKT and CCND1 expression in MDA-MB-436 cells.

Supplementary Figure 8

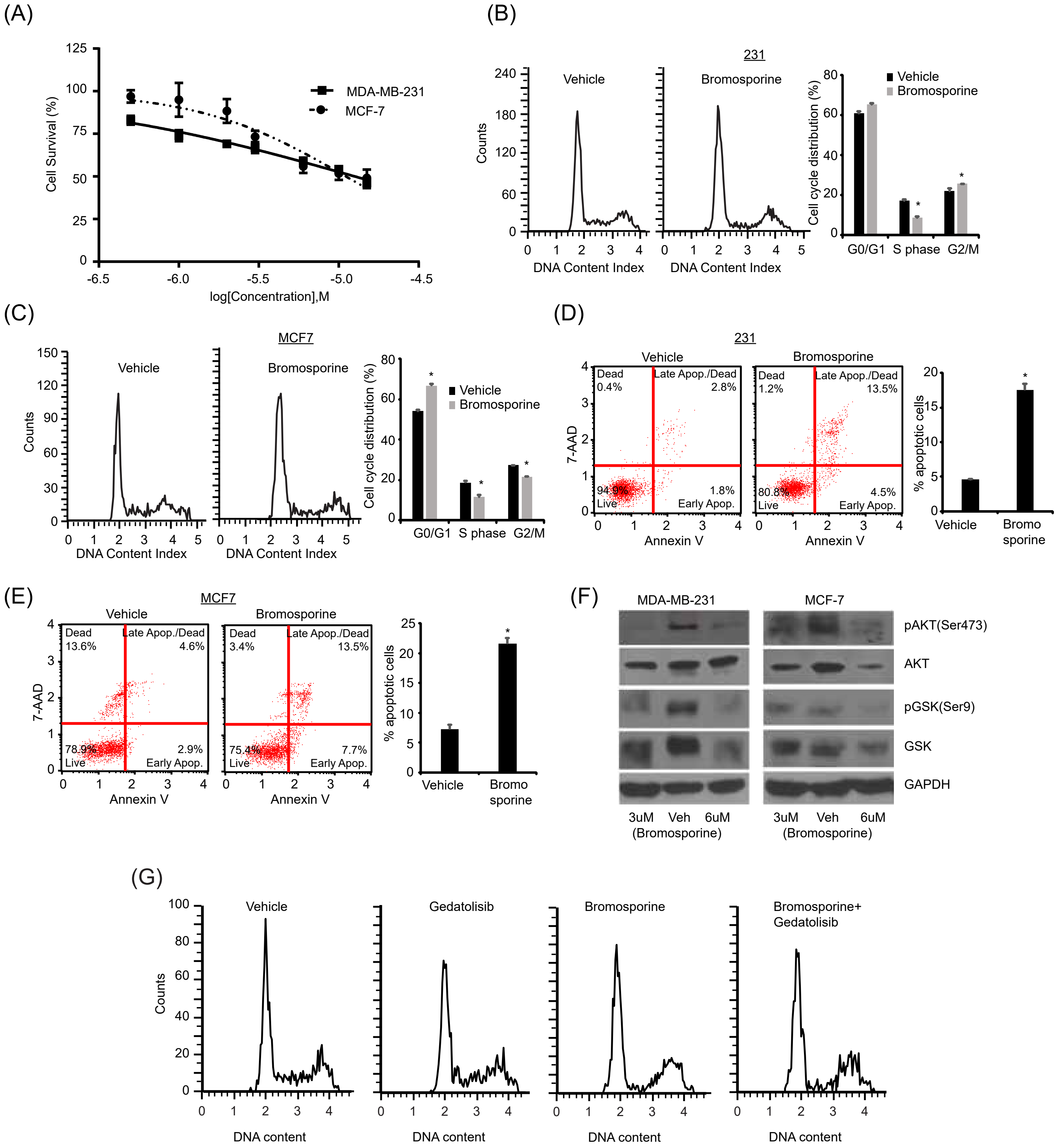

Supplementary Figure 8. Bromosporine treatment of MDA-MB-231 and MCF-7. (A) Survival curves of 231 and MCF-7 cells following treatment with bromosporine. (B and C) Cell cycle analysis following treatment of 231 (B) and MCF7 (C) cells. (D and E) Analysis of apoptotic rate after treatment of 231 (D) and MCF7 (E) cells based on detection of 7-AAD and Annexin V. (F) Western analysis of expression of different proteins following treatment of 231 and MCF7 cells. (G) Cell cycle profiles of MDA-MB-231 cells following treatment with gedatolisib, bromosporine or combination of the two. * $p < 0.05$.

Table S1. FISH results as counts of signals from BAC clones mapping the BPTF locus and the centromeric probe for chromosome 17 (CEP17).

Core ID	BPTF mean copy number	BPTF STDEV	CEP 17 mean copy number	CEP17 STDEV	BPTF/CEP17 Ratio
AA1	3.8	2.0	3.8	1.4	1.0
AA2	3.5	1.6	3.4	1.8	1.0
AA4	2.2	0.9	2.2	0.8	1.0
AA5	2.5	1.1	2.5	1.1	1.0
AA8	1.8	0.5	2.0	0.7	0.9
AB1	2.1	0.5	1.8	0.7	1.2
AB2	3.0	1.1	3.2	1.1	0.9
AB4	1.8	0.4	1.8	0.4	1.0
AB8	2.1	0.7	2.1	0.7	1.0
AC2	3.8	1.4	3.6	1.4	1.1
AC4	2.4	0.7	1.2	0.5	2.0
AC5	1.8	0.5	2.1	0.5	0.9
AC6	2.2	0.9	2.3	0.9	1.0
AC7	4.8	1.8	4.7	1.6	1.0
AC8	2.0	0.7	2.1	0.7	0.9
AD1	3.0	2.6	2.9	2.9	1.0
AD5	2.3	1.0	2.5	0.9	0.9
AD6	3.5	1.3	3.3	1.3	1.1
AD7	3.1	1.3	3.1	1.7	1.0
AE1	3.3	1.4	2.1	0.7	1.5
AE4	3.0	2.1	2.1	1.6	1.5
AE5	3.1	1.1	3.1	1.1	1.0
AE7	2.7	1.0	1.9	0.9	1.4
AF4	1.9	0.5	2.0	0.6	1.0
AF5	1.8	0.4	1.8	0.5	1.0
BA1	2.1	0.7	2.3	0.9	0.9
BA4	1.9	0.7	2.1	0.8	0.9
BA6	2.5	1.0	2.6	1.1	1.0
BA7	3.4	1.8	2.1	1.4	1.6
BA8	2.0	0.6	2.0	0.7	1.0
BB2	2.0	0.6	1.8	0.4	1.1
BB3	2.0	0.5	2.0	0.8	1.0
BB4	4.7	1.9	5.1	2.5	0.9
BB6	2.8	1.4	2.0	0.7	1.4
BB7	1.9	0.4	2.0	0.6	0.9
BB8	3.2	1.2	3.1	1.2	1.0
BC1	2.9	1.4	3.1	1.3	0.9

BC3	4.1	2.6	4.0	2.6	1.0
BC4	2.1	0.9	2.3	1.0	0.9
BC5	1.8	0.4	1.8	0.4	1.0
BC7	2.4	1.1	2.5	1.0	1.0
BC8	5.5	2.5	5.3	2.4	1.0
BD4	3.6	1.3	2.1	1.0	1.7
BD5	20.0	0.0	2.6	1.0	7.8
BD6	2.4	1.0	1.6	0.6	1.5
BD8	2.0	0.7	2.0	0.6	1.0
BE1	20.0	0.0	2.4	1.2	8.2
BE4	3.3	1.7	3.5	1.7	0.9
BF1	1.9	0.4	1.8	0.4	1.0
BF5	4.6	2.5	4.2	2.4	1.1
CA1	20.0	0.0	3.1	1.5	6.5
CA2	2.7	1.6	2.7	1.7	1.0
CA4	2.1	0.7	1.9	0.5	1.1
CA5	2.1	0.8	2.1	0.6	1.0
CB1	2.8	1.3	2.8	1.4	1.0
CB3	2.2	0.9	2.5	1.1	0.9
CB4	2.2	0.8	2.4	0.9	0.9
CB5	20.0	0.0	2.4	1.3	8.2
CB6	3.0	1.4	2.6	1.3	1.1
CB7	3.0	1.4	3.1	1.5	1.0
CB8	2.7	1.1	2.7	1.1	1.0
CC2	2.1	0.8	2.1	0.7	1.0
CC3	1.9	0.6	2.0	0.6	1.0
CC6	5.2	3.8	2.5	1.1	2.0
CC8	2.4	1.0	2.4	1.0	1.0
CD2	2.3	0.9	2.5	1.0	1.0
CD6	2.1	0.8	2.2	0.7	1.0
CE5	1.8	0.4	1.8	0.4	1.0
CE8	7.8	6.7	4.0	2.1	2.0
CF6	2.1	0.8	2.1	0.7	1.0
CF8	1.8	0.4	1.9	0.3	1.0
DA1	20.0	0.0	3.5	2.2	5.7
DA3	3.7	1.8	2.3	1.1	1.6
DB1	3.7	1.8	3.3	1.9	1.1
DD4	2.0	0.6	1.9	0.6	1.0
DD5	7.6	4.7	2.9	1.5	2.6
DD8	2.0	0.7	2.2	1.8	0.9
DE2	2.4	1.0	2.5	0.9	0.9
DE4	1.9	0.5	1.9	0.5	1.0
DE6	2.9	1.4	2.6	1.5	1.1

DE7	3.0	1.0	1.5	0.6	2.0
DE8	1.9	0.4	1.9	0.5	1.0
DF1	2.7	1.0	2.4	0.9	1.1
DF3	2.3	0.9	2.5	0.9	0.9
DF6	1.9	0.4	1.9	0.6	1.0
